# Evaluation of fracture behavior in short fiber–reinforced direct and indirect overlay restorations

**DOI:** 10.1007/s00784-023-05164-2

**Published:** 2023-07-21

**Authors:** S. Garoushi, A. Ö. Akbaşak-Sungur, S. Erkut, P. K. Vallittu, S. Uctasli, L. Lassila

**Affiliations:** 1 Department of Biomaterials Science and Turku Clinical Biomaterial Center -TCBC, Institute of Dentistry, University of Turku, Turku, Finland; 2 Department of Prosthetic Dentistry, Faculty of Dentistry, Baskent University, Ankara, Turkey; 3 Wellbeing Services County of Southwest Finland, Turku, Finland; 4 Department of Prosthodontics, Faculty of Dentistry, University of Ankara, Ankara, Turkey

**Keywords:** Fracture resistance, Bilayered restoration, CAD/CAM, Overlays, MOD, Short-fiber composite

## Abstract

**Objectives:**

The aim was to assess how incorporating a short-fiber composite (SFC) core would affect the fracture behavior of direct and indirect overlays. Furthermore, to examine the relationship between the thickness ratio of SFC core to particulate-filled composite (PFC) veneering and the fracture-behavior of bilayered-structured restorations.

**Materials and methods:**

A total of 120 molars were used to create MOD cavities, with palatal cusps removed. Four different groups of direct overlays were then made (*n* = 15/group), all of which featured a SFC core (everX Flow) with varying thicknesses (0, 1, 4, and 5 mm), as well as a surface layer of PFC (G-aenial Posterior), with the overall thickness of the bilayered-structured restoration set at 5 mm. Additionally, four groups of CAD/CAM restorations were created (Cerasmart 270 and Initial LiSi Block), with or without 2 mm of SFC core reinforcement. Following the fabrication of these restorations, cyclic fatigue aging was carried out for a total of 500,000 cycles, with an applied maximum load (*F*_max_) of 150 N. Subsequently, each restoration underwent quasi-static loading until fracture. The fracture mode was subsequently evaluated using optical microscopy and SEM.

**Results:**

There were no statistically significant differences (*p* > 0.05) observed in the fracture resistance of indirect overlays reinforced with a 2-mm SFC core compared to those made solely from restorative materials. Direct overlays constructed using plain SFC or with a 4-mm layer thickness of SFC core exhibited significantly higher fracture resistance values (2674 ± 465 and 2537 ± 561 N) (*p* < 0.05) when compared to all other groups tested, according to the statistical analysis ANOVA.

**Conclusions:**

The most effective method for restoring large MOD cavities was found to be direct restoration using SFC either alone or as a bulk core in combination with PFC composite.

**Clinical relevance:**

The use of SFC as bulk reinforcing base will significantly improve the loading performance of directly layered restorations.

## Introduction

In clinical practice, it is common to face the challenge of restoring posterior teeth that have considerable damage to their crown. Thanks to the recent advancements in adhesive dentistry, numerous treatment options that are minimally invasive have become accessible. The preservation of the remaining tooth structure is crucial in order to support the restoration, and it is considered a significant factor when choosing alternative treatment methods. To avoid removing healthy tooth structure, adhesive ceramic overlay restorations have been used instead of full-coverage restorations. With the advancement of modern materials, fabrication techniques for ceramic restorations have shifted from conventional hand-layering to computer-aided design and manufacturing (CAD/CAM) technology [1]. However, all-ceramic restorations do possess certain drawbacks. These include their inherent brittleness, higher cost compared to other options, the need for more extensive tooth reduction during preparation, potential wear on opposing teeth, and the complex bonding process that can increase chairside time [1–3]. Restorations made of resin composite can be a cost-effective substitute for full ceramic restorations. Compared to ceramics, composites are relatively easier to construct and may cause less wear on opposing teeth [4, 5].

Restorations made with direct resin composite do not need a special kind of preparation. These restorations can be completed in a single treatment session and are relatively cost-effective. In vitro studies have shown that direct resin composite restorations are associated with shrinkage stress due to polymerization [6, 7]. However, there is currently no direct clinical evidence to suggest any harmful effects caused by these stresses [8]. On the other hand, indirect composite restorations, fabricated in the laboratory, are recommended for large cavities as a solution to address issues associated with polymerization kinetics and shrinkage stress. These restorations are believed to facilitate a more accurate restoration of the original morphology. Regrettably, there is a lack of studies in the literature that directly compare the survival rates of direct and indirect composite restorations. In a recent retrospective long-term follow-up study, the authors found no statistically significant difference in survival rates between direct and indirect composite cusp-replacing restorations [9]. Therefore, both direct and indirect resin composite restorations are considered suitable treatment options. However, considering the longer treatment time and higher costs associated with indirect restorations, the direct technique is favored [9].

In 2022, Heintze et al. carried out a meta-analysis on the clinical efficacy of resin-based direct posterior restorations [10]. They identified the principal reasons for the restoration replacement were bulk fractures and wear, which accounted for about 70% of replacements.

This bulk fracture failure is highlighting the importance of fracture toughness as a critical characteristic for achieving satisfactory clinical outcomes. As per the literature, particulate-filled resin composite (PFC) materials are still not considered suitable for high-stress-bearing areas due to their insufficient toughness [3, 11]. Therefore, it is not clear whether PFCs should be used in clinical applications involving high-stress bearings such as large MOD (mesio-occlusal-distal) or posterior overlay restorations, given their potential for failure. There has been extensive research aimed at developing a method to strengthen the remaining tooth structure and large composite restorations. One way to support the use of resin composite in complex clinical scenarios is by using short fiber–reinforced composite (SFC). This is achieved by combining short glass fibers with the filler structure to improve its resistance to crack propagation. This technology has seen significant advancements and has been studied in various research works [11–14]. Researchers have tried to utilize SFC material as a reinforcing structure beneath the surface or veneer layer of conventional PFC material, resulting in the creation of bilayered-structured composite restorations [15, 16]. Laboratory studies have demonstrated that teeth restored with this bilayered-structured system had a higher capacity for bearing loads and a more favorable mode of fracture [17–21]. These studies have shown that SFC can reinforce both the remaining tooth structure and the composite restoration by acting as a foundation that prevents cracks from propagating even in the tooth structure [18, 19].

As far as we are aware, there has not been comprehensive research on the use of SFC as a supporting structure beneath CAD/CAM-fabricated restorations. Although there is ample information available on the properties of SFC or veneering material [22, 23], little is known about the loading behavior of the material combination. A previous study by authors showed promising quasi-static fracture behavior results for bilayered-structured direct/indirect restorations integrating SFC core and conventional surface material [20], but no information is available on the fracture behavior after prolonged cyclic fatigue aging. Therefore, the purpose of this study was to examine how SFC core impacts the fracture behavior of various direct/indirect posterior overlays following fatigue aging. Additionally, the study aimed to assess how the fracture behavior of bilayered composite restorations is affected by the ratio of the thickness of the SFC core layer to the thickness of the veneering PFC layer.

## Materials and methods

The composition of the materials utilized in this research is presented in Table 1. A total of 120 extracted mandibular molar teeth were carefully chosen for this study, ensuring that they exhibited no evidence of occlusal wear or dental caries. Teeth were selected based on similar occlusal size. After the removal of soft tissues under tap water, the teeth were preserved in a 0.5% chloramine T solution at 4 °C for no more than 2 months. The measurements of each tooth’s dimensions were conducted using a digital caliper. The average dimensions obtained were consistent with our earlier measurements, with values of 10.5 mm (± 0.5 mm) and 11.5 mm (± 0.6 mm) for the bucco-lingual and mesio-distal directions, respectively. The methodology for mounting the teeth on an acrylic block with a diameter of 2.5 cm using auto-polymerized acrylic resin (Palapress; Heraus Kulzer, Wehrheim, Germany) was consistent with our earlier study. Similarly, a standardized coronal preparation was performed by the same operator who was responsible for both the preparations and the subsequent restorations, ensuring consistency across the experiments.

**Table 1:** The restorative materials used in the study

Material (code)	Manufacturer	Composition
G-aenial Posterior (PFC)	GC Corp, Tokyo, Japan	UDMA, dimethacrylate co-monomers, prepolymerized silica and strontium fluoride containing fillers 77 wt%
Cerasmart 270 (CS270)	GC Corp, Tokyo, Japan	Bis-MEPP, UDMA, dimethacrylate, silica (20 nm), barium glass (300 nm) 71 wt%
Initial LiSi Block (LiSi)	GC Corp, Tokyo, Japan	Lithium disilicate glass ceramic
everX Flow (SFC) Bulk shade	GC Corp, Tokyo, Japan	Bis-EMA, TEGDMA, UDMA, short glass fiber (200–300 µm and Ø7 μm), barium glass 70 wt%

*TEGDMA*, triethylene glycol dimethacrylate; *UDMA*, urethane dimethacrylate; *Bis-MEPP*, bis(p-methacryloxy (ethoxy)1–2 phenyl)-propane; *Bis-EMA*, ethoxylated bisphenol-A-dimethacrylate; *wt%*, weight percentage

### Preparation of teeth and restorative techniques

**Fig. 1:**
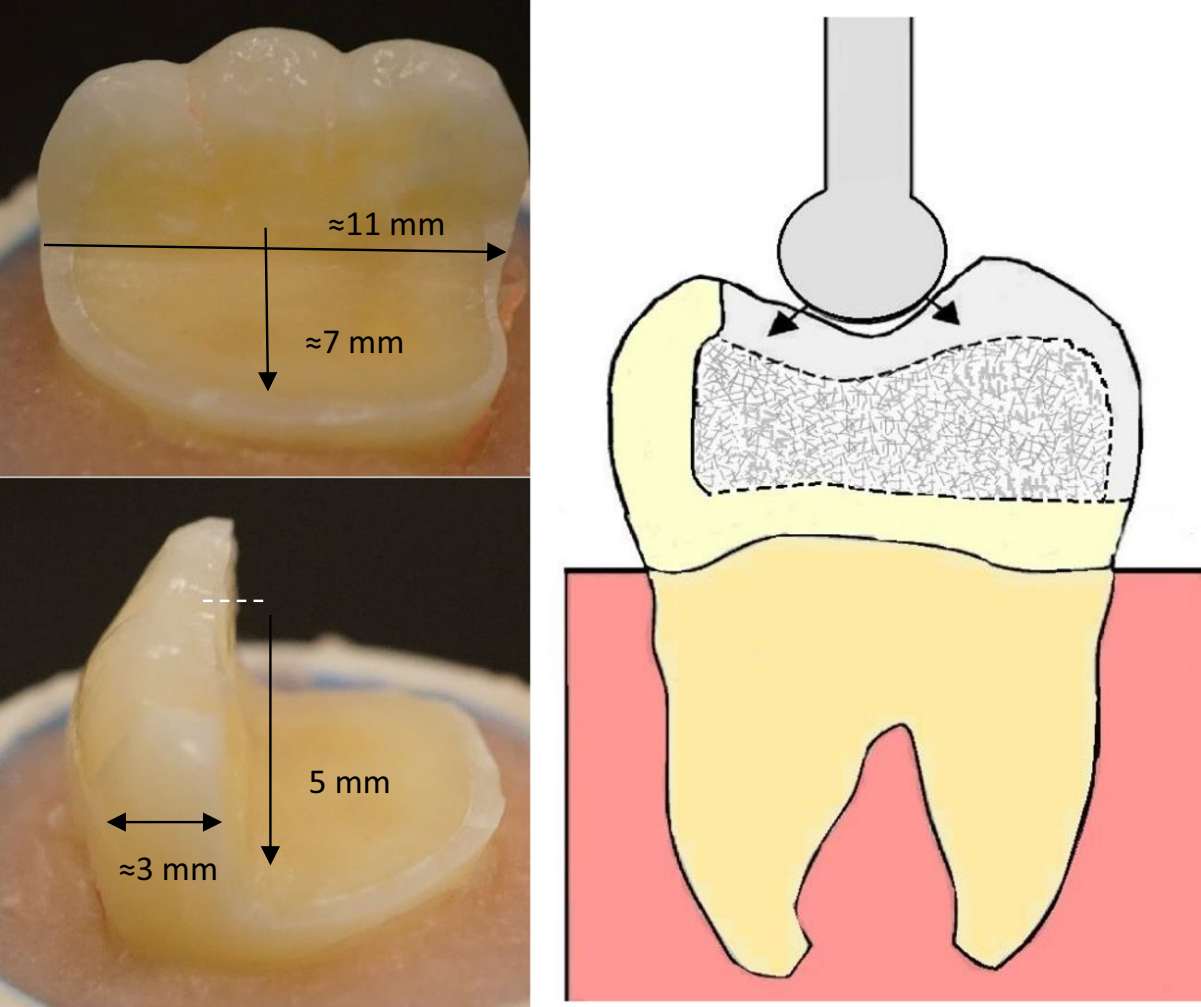
A photograph and schematic drawing representing tooth preparation measurements in millimeters, bilayered structure restoration and the load test setup. Modified from previous study [20]

The preparation and restoration adopted in this study follow the same methodology employed in our previous study [20]. Standardized tooth preparations were performed to simulate large MOD cavities with the lingual cusps removed. The measurements were obtained using a periodontal probe and standard burs to ensure accuracy of the dimensions. The preparations were carried out using a high-speed handpiece equipped with flat-end parallel carbide (H21LR.314.010, Brasseler, Savannah, GA, USA) and round-end diamond burs (850–014 M SSWhite, Lakewood, NJ, USA) with water cooling. The cavity floor was flat, and the occlusal reduction measured 5 mm, while the remaining buccal wall thickness averaged around 3 mm (Fig. 1). The margins of the restorations were positioned approximately 1–1.5 mm above the cement-enamel junction (CEJ). Following the cavity preparation, all specimens underwent the same adhesive protocol described in our previous studies [20, 21]. The tooth surfaces were prepared for bonding with a selective acid-etching (37% phosphoric acid) and with adhesive using one-bottle universal bonding agent (G-Premio Bond, GC Corp., Tokyo, Japan) according to the manufacturers’ instructions. Two different approaches, namely, direct and indirect restorations, were employed to mimic clinical techniques for the tooth restorations. Sample size was selected based on previous fatigue studies from literature [16, 17, 19, 21].

### Direct restoration

In the direct restoration group, standardized restorations were created using a translucent model of the tooth crown prior to the preparation process. This approach ensured consistency and standardization in the restorations. To investigate the impact of the thickness ratio between the SFC core and the veneering direct composite (PFC), different groups (Table 2) were established. The SFC cores were varied in thickness, ranging from 0, 1, 4, to 5 mm. Precise control over the SFC core thickness was achieved by horizontally applying the material onto the cavity floor, utilizing a scaled periodontal probe. The following groups (*n* = 15/group) were created: PFC group (0-mm SFC core + 5-mm PFC), PFC + 1SFC group (1-mm SFC core + 4-mm PFC), PFC + 4SFC group (4-mm SFC core + 1-mm PFC), and SFC group (5-mm SFC core + 0-mm PFC).

**Table 2:** Restorative groups with different structures (*n* = 15/group)

Group	Restorative approach	Restoration structure
PFC	Direct	Monolayered structured
PFC + 1SFC	Direct with 1-mm core of SFC	Bilayered structured
PFC + 4SFC	Direct with 4-mm core of SFC	Bilayered structured
SFC	Direct	Monolayered structured
CS270	Indirect	Monolayered structured
CS270 + SFC	Indirect with 2-mm core of SFC	Bilayered structured
LiSi	Indirect	Monolayered structured
LiSi + SFC	Indirect with 2-mm core of SFC	Bilayered structured

The direct composite restorations were created by manually layering the PFC composite (G-aenial Posterior) into the space between the index and the prepared cavity, with or without the inclusion of the SFC core. Each layer was cured for 40 s from all directions using a handheld light curing device (Elipar TM S10, 3 M ESPE, Seefeld, Germany) emitting a wavelength of 430–480 nm and a light intensity of 1600 mW/cm^2^. The light curing tip was positioned in close proximity (1–2 mm) to the resin composite surface. To compensate for the missing axial walls in all groups, a 1-mm layer of PFC was applied and built up. All groups’ missing axial walls were built up using PFC composite (1 mm).

### CAD/CAM restoration

The indirect restorations (*n* = 15/group) were made using either Cerasmart 270 or LiSi blocks, with or without SFC as a core material (Table 2). In bilayered structure groups, the SFC core was 2-mm thick, leaving a space of 3 mm occlusally, 1 mm proximally, and lingually for the veneering CAD/CAM materials. For these specific groups (after application of SFC), a photoimpression of the prepared cavity was captured, and the restoration was designed and milled using CEREC technology (Sirona Dental Systems Inc., Long Island City, NY). Prior to luting, the fitting (inner) surface of all overlays was treated with acid-etching using hydrofluoric acid (5%, IPS Ceramic Etching Gel, Ivoclar, Schaan, Liechtenstein) for 60 s, followed by thorough washing and air-drying. Subsequently, the overlays were luted using G-Multi Primer (GC Corp) and dual-cure resin cement (G-CEM LinkForce, GC Corp). Light curing was performed using a hand-light curing unit (Elipar TM S10) for 20 s per segment. Before testing, all overlays underwent polishing with abrasive polishing points and were then stored in water at 37 °C for a duration of 2 weeks.

### Fracture load test

In contrast to our previous study [19], an additional step was made before the quasi-static fracture load test. Prior to this test, the restored teeth underwent cyclic fatigue aging. The specimens were immersed in a 37 °C water bath within a chewing simulator (MOD, Esetron Smart Robotechnologies, Ankara, Turkey). Subsequently, mechanical dynamic loading was applied to the specimens, subjecting them to 500,000 cycles. The loading was performed at a frequency of 1.5 Hz and a force of *F*_max_ = 150 N. The total water immersion time of specimens during and after cyclic aging was 4 weeks. After the cyclic fatigue aging process, a quasi-static load was applied directly to the specimens in each group (*n* = 15 per group). This was accomplished using a universal testing machine (Lloyd model LRX, Lloyd Instruments Ltd, Fareham, UK) at a speed of 1 mm/min. A metal ball with a diameter of 5 mm was used to deliver the load vertically between the triangular ridges of the lingual and buccal cusps (as shown in Fig. 1). The loading curve was closely monitored until the point of restoration fracture, which was indicated by the final incline in the load–deflection curve.

The researchers visually inspected the fracture modes of each loaded restoration and classified them into two types: catastrophic fracture that affects both the restoration and tooth structure, and fracture of only the restoration.

### Fracture mode analysis

The fracture mode of the restorations was assessed through visual examination and under a stereomicroscope at various magnifications and illumination angles (Heerbrugg M3Z, Heerbrugg, Switzerland). Three independent researchers examined the specimens and reached a consensus on the type, position, and direction of failure. To gain further insights into the fractures, representative specimens were analyzed using scanning electron microscopy (SEM) with a focus on the upper loading surfaces and inner structures. Using a vacuum evaporator and a sputter coater (BAL-TEC SCD 050 Sputter Coater, Balzers, Liechtenstein), all specimens were coated with a layer of gold prior to the observation.

### Statistical analysis

To compare the impact of different restorative procedures on the load-bearing resistance of the restorations, analysis of variance (ANOVA) at the *p* < 0.05 significance level was employed followed by post hoc log rank test. The significance level was set at 0.05. Statistical analysis was conducted using SPSS software version 23 (SPSS Inc., Chicago, IL).

## Results

**Fig. 2:**
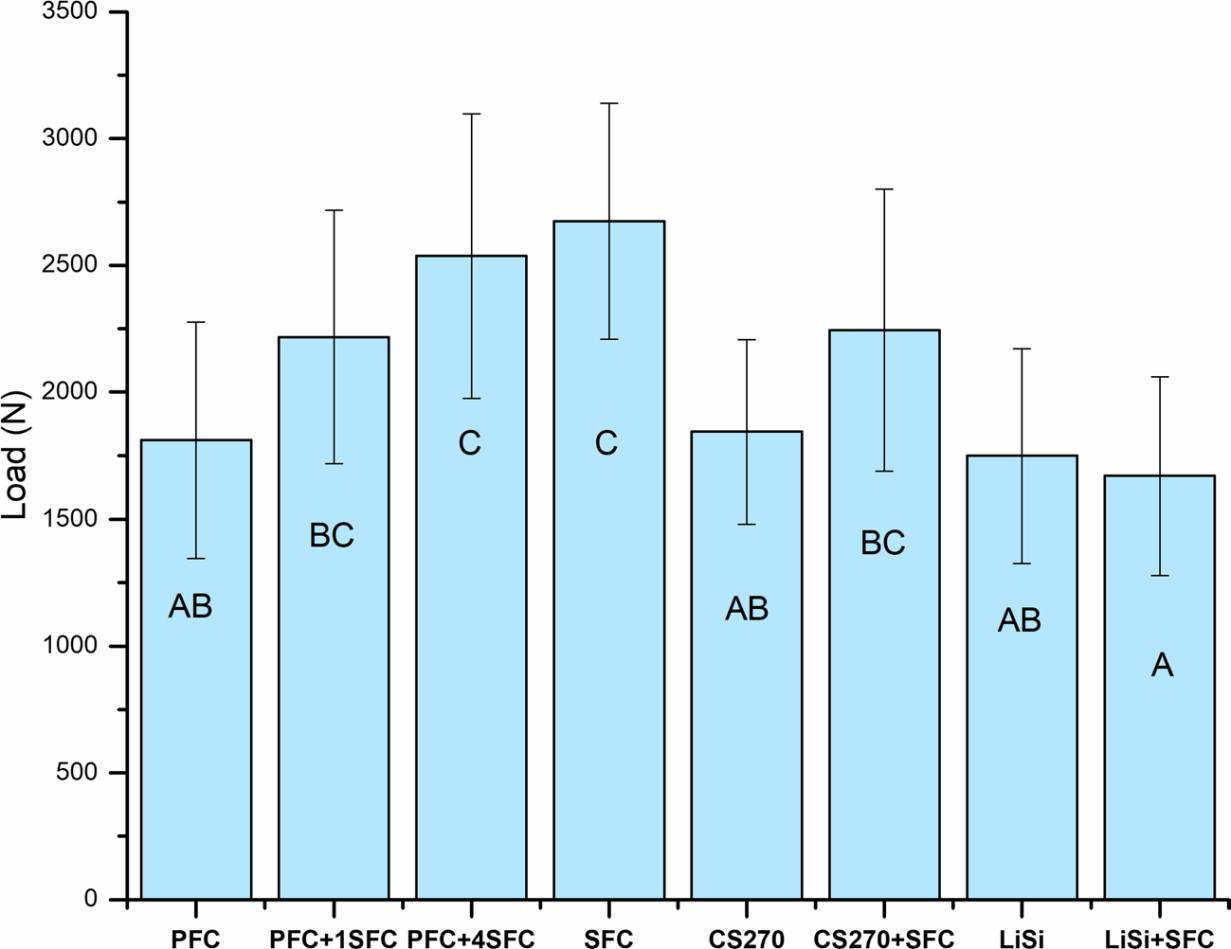
Mean values of load-bearing capacity (N) and standard deviation (SD) of tested restorations (monolayered/bilayered structure). The same letters inside the bars represent non-statistically significant differences (*p* > 0.05) among the materials

**Fig. 3:**
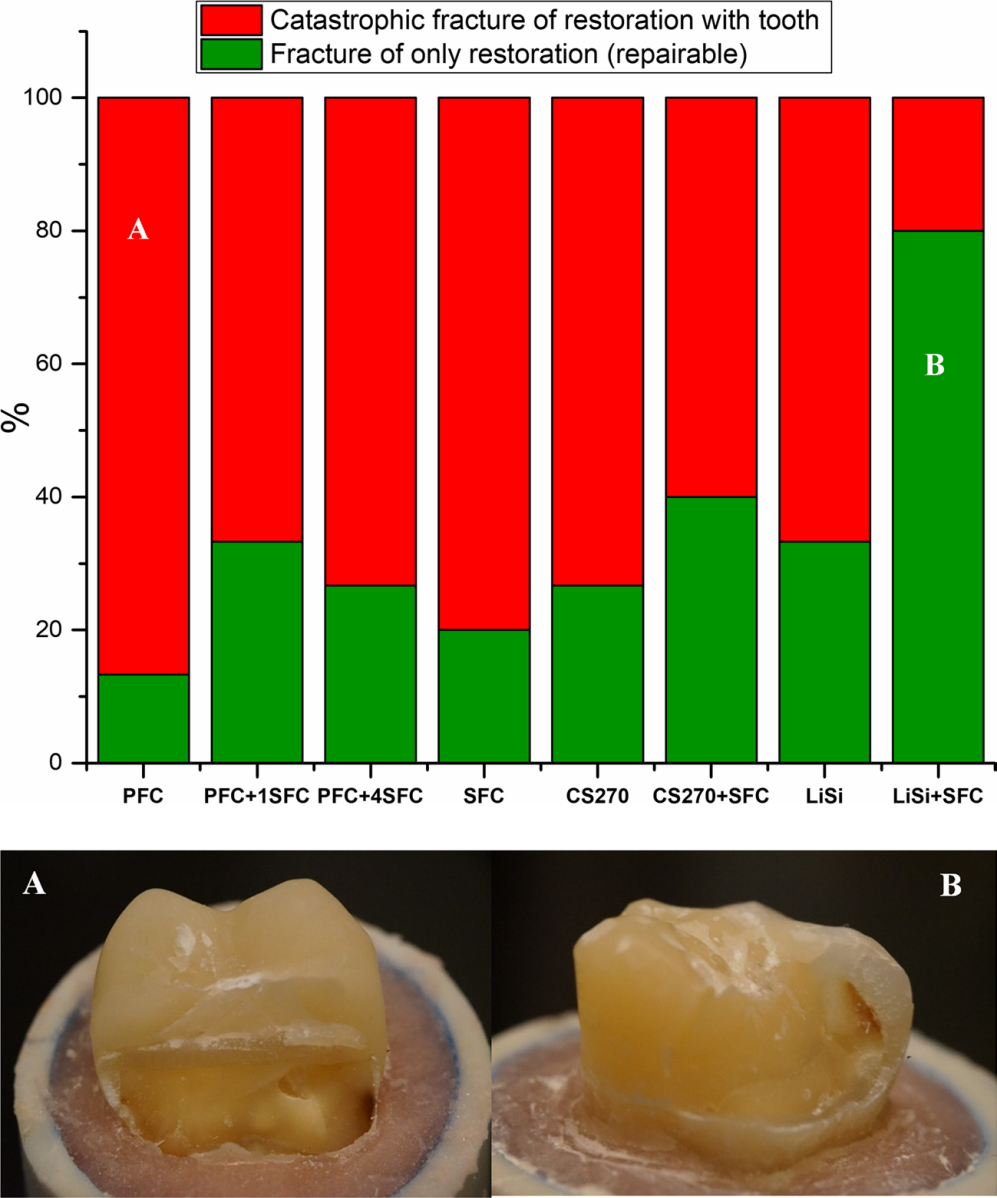
Percentage and photographs of various fracture modes of tested restorations with and without SFC core

**Fig. 4:**
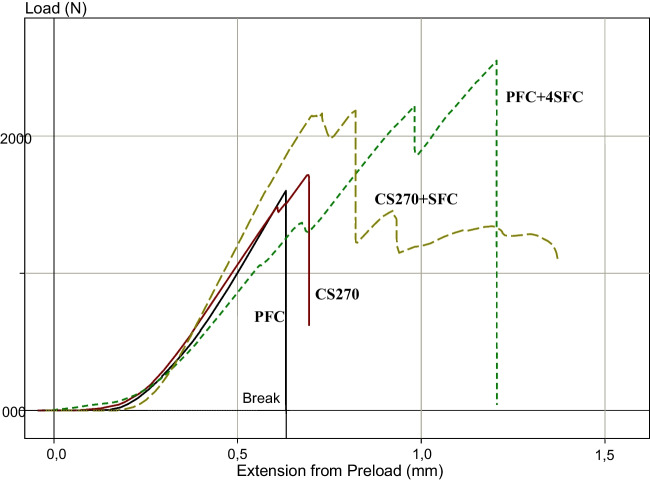
Load–deflection curve of representative specimens from direct and indirect restoration groups with and without SFC core

All of the restorations remained intact throughout the cyclic fatigue aging period, and as a result, all of the fatigued specimens were loaded to failure under quasi-static loading. Figure 2 displays the average fracture load values of the restorations following cyclic fatigue aging. The results of the ANOVA analysis revealed that there was a significant increase in load-bearing capacities for direct restorations when reinforced with thick SFC core (4 and 5 mm) (2537 ± 561 and 2674 ± 465 N) (*p* < 0.05) compared to other tested direct and indirect overlays (in the range between 1670 and 2243 N). On the other hand, there were no statistically significant changes (*p* > 0.05) in the load-bearing capacities between restorations reinforced by a 2-mm SFC core (bilayered structured) using the indirect technique (CS270 + SFC:2243 and LiSi + SFC:1670 N) and those made from plain (monolayered structured) restorative materials (1844 ± 360 and 1749 ± 423 N). Even though significant loading forces were applied, none of the restorations exhibited adhesive failure. Upon visual inspection of the specimens, it was observed that there were two distinct types of fractures, as depicted in Fig. 3. The predominant fracture type observed in the specimens with SFC core, as shown in Fig. 3, was the fracture of only the restoration. This type of failure is repairable. On the other hand, the fracture involving both the restoration and tooth structure, extending to the root, was mostly observed in the monolayered structured specimens. This type of fracture is considered unrepairable. Figure 4 shows typical load–deflection curves of representative specimens from direct and indirect restoration groups with monolayered or bilayered structure. Restorations that were reinforced by SFC core exhibited a significant rise in their ability to withstand load before fracturing.

**Fig. 5:**
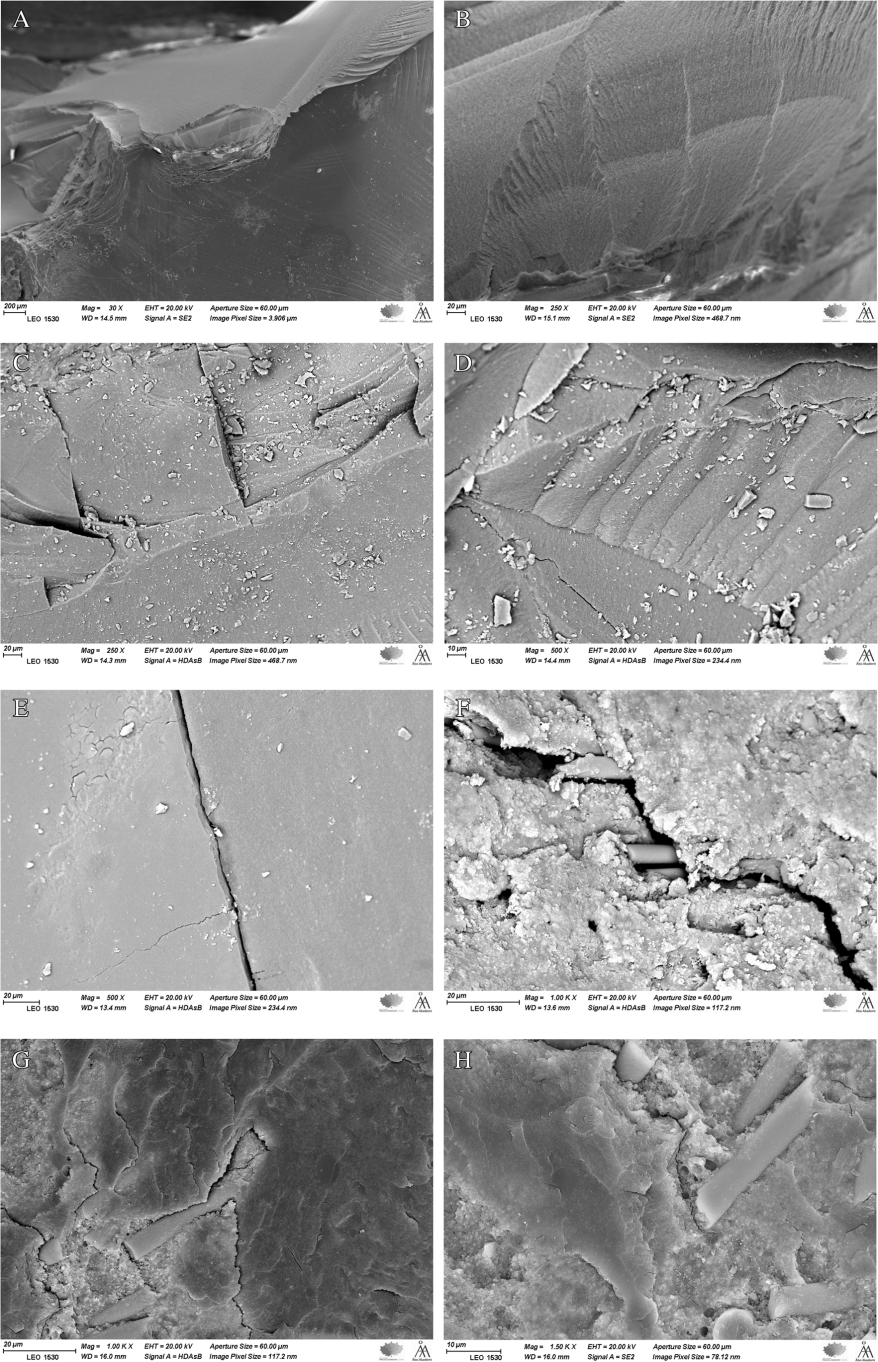
SEM images of fracture surfaces of investigated bilayered structure restorations observed under different magnifications showing arrest lines with twist hackles (**A**–**D**, arrows) and a radial cracks (**D** & **E**) propagated through the restoration from the load application area to the SFC core. **F**, **G**, and **H** show short fibers’ ability to re-direct and hinder crack propagation (arrows)

Figure 5 displays SEM images that are representative of fractured restorations. The images, captured at various magnifications, represent several fracture markers, such as arrest lines. These arrest lines consist of multiple concave lines that indicate the downward radial propagation of the crack. Additionally, the images reveal the presence of fine twist hackles, which originate between the arrest lines. Furthermore, the images demonstrate the initial spread of the crack line before being deflected and hindered by the short fibers of the SFC-core.

## Discussion

In this study, the impact of two restorative approaches (monolayered or bilayered structure with SFC core) utilizing various direct/indirect restorative materials on the fracture behavior of large MOD restorations was investigated after extended cyclic fatigue aging of 500,000 cycles. The preparation and restoration techniques employed in the study aimed to replicate scenarios involving substantial loss of tooth structure, which could be restored through either direct or indirect methods, in accordance with our previous study [20]. The results of our research revealed variations in the load-bearing capacity of the restorative techniques employed when SFC-core was used (as illustrated in Figs. 2 and 3). However, the application of SFC core in a thin layer of 1–2 mm did not yield any statistically significant difference in the outcomes. The findings of our study are consistent with previous research which suggested that adding SFC as a base material in the cavities of posterior teeth that were restored with thick conventional overlays did not improve their fracture behavior [16, 24–26]. On the contrary, alternative research has indicated that cavities were repaired using a bilayered composite approach, incorporating SFC as a bulk core material, exhibited superior load bearing capability, and exhibited a favorable fracture pattern [27–30]. The variations observed in the outcomes of these studies can be owed to a number of variables, including variations in the experimental setup, variations in the adhesive strategy used, and variations in the thickness ratio between the SFC core composite and overlay material. Our findings align with this explanation, as our data demonstrated that restorations utilizing a thick SFC core (measuring 4–5 mm in thickness) exhibited significant enhancements in load-bearing capacity compared to those utilizing plain PFC (Fig. 2).

The function of the SFC core is to provide support to the veneering layer and serve as a layer that can stop cracks from propagating further [11, 12]. In order to effectively reinforce the veneered material, the integral toughness of the SFC core should be greater than that of the veneered layer [17]. The orientation of fibers as well as the density of cross-linking within the polymer matrix may play a crucial role in achieving this. If the SFC core is to serve as a crack-stopper, it is also critical to consider the distance between the point where stress is initiated on the surface and the SFC core. Therefore, the thickness of the veneered material may affect its load-bearing capacity and ability to propagate cracks. Previous research has also highlighted the importance of properly applying the SFC and veneered layers, particularly with regard to their thickness [30].

The currently used PFC composite, hybrid ceramic, and lithium disilicate glass ceramic materials are inherently strong but brittle, requiring additional toughness [31]. One major issue with using brittle materials to restore missing dentine is their substantially lower fracture toughness compared to that of natural dentine [12]. This concern becomes even more pronounced in larger restorations where the volume of brittle material is greater [32]. Thus, direct or indirect composite or ceramic restorations may not be the optimal choice when there is a significant loss of tooth structure due to this shortcoming.

As previously mentioned, the fracture toughness property is used to measure the ability of brittle materials to prevent crack growth when they are under load, making it an important predictor of structural performance and fatigue durability [11]. Previous investigations have demonstrated that the flowable SFC material (everX Flow) utilized in this study exhibits notable values of flexural strength and fracture toughness [17, 22, 33]. To the best of our knowledge, there are no other dental composites that exhibit fracture toughness values comparable to the 2.6 MPa m^1/2^ demonstrated by everX Flow. Contrarily, the stated values for fracture toughness for various direct and indirect restorative materials, including ceramic and composite, are typically in the range of 1.1 to 1.9 MPa m^1/2^ [34, 35].

Despite the manufacturer’s recommendation to exclusively use flowable SFC to replace lost dentine for both direct and indirect restorations, as previously mentioned, the study’s results showed that flowable SFC alone had the highest load-bearing capability (Fig. 2). This approach has been a trend in recent studies, aimed at providing a stronger and more durable solution by maximizing the amount of fibers to rebuild the entire missing structure [17, 36].

Restoration specimens that had only a veneering material and no fiber reinforcement showed a type of fracture that was catastrophic and impossible to repair, as illustrated in Fig. 3. According to Chai’s findings, this type of fracture appeared to be caused by median-radial cracks that extended from the point of loading into the material [37]. It was evident that the veneering material’s brittleness led to this type of catastrophic fracture. However, when a SFC core was used (in bilayered structured restorations) instead of plain veneering material (in monolayered structured restorations), the fracture mode shifted to become more repairable. Although this behavior was not as evident as in some previous studies [15–17], it could be due to the effects of prolonged cyclic fatigue aging.

SEM images demonstrate the presence of crack lines originating from the loading area and extending toward the deeper structure. Fractographic markers, such as twist hackle and arrest lines, are observable in the images (Fig. 5). Notably, arrest lines are particularly valuable in determining the direction of crack propagation, as they typically initiate on the concave side of the initial arrest line, as noted by Scherrer and colleagues [38]. In contrast, fiber-reinforced composites demonstrated the capability to alter and block the spread of cracks within the materials. As illustrated in Fig. 5, the existence of these fibers, which can absorb energy and distribute stress, enables the deflection of crack propagation away from the core of the material.

Even when exposed to forces higher than typical masticatory forces, none of the restorations, whether direct or indirect, showed signs of adhesive failure, indicating the effectiveness of the bonding. The bonding of luting resin to CAD/CAM material is likely a result of a combination of chemical bonding facilitated by a primer and micromechanical retention achieved through acid etching.

It is noteworthy that when comparing the data obtained from this current study to our previous research [20] that utilized the same standardized restorative technique but did not include cyclic fatigue aging, the load-bearing values fell within the same range. This indicates that the restorations tested in the study were not weakened by low-load/high-cycle (500,000 cycles) fatigue testing. This is why some researchers [16, 28, 36] prefer to use an accelerated fatigue setup rather than a true fatigue test, which was the method used in this study.

In direct resin composites and CAD/CAM blocks, mechanical degradation mechanisms have been linked to the development of microcracks in cyclically aged specimens [39, 40]. These microcracks could serve as crucial faults in materials with relatively high brittleness, reducing the materials’ strength as well as their reliability. On the other hand, Tiu et al. demonstrated that the toughening behavior of fiber-reinforced resin composites was unaffected by aging and consequential hydrolysis [41]. They contend that in the absence of strong fiber-matrix adhesion, the mechanism of fiber bridging is completely functional.

From a practical standpoint, it is clear that the morphology and occlusion of restorations can be better controlled using indirect overlay techniques, rather than direct methods. However, the economic feasibility of such treatments may be limited for some patients. Clinical trials are necessary to verify the effectiveness of bilayered-structured restorations that utilize SFC core as a dentine substitute.

It is important to consider the limitations of this study, such as the absence of thermal aging and long-term water storage, as well as the use of other established restorative CAD/CAM materials such as monolithic zirconia. Another limitation was the lack of periodontal ligament simulation. However, this in vitro study offered a high degree of standardization by controlling tooth and preparation dimensions, loading conditions, and occlusal morphology.

## Conclusion

Within the limitations of this study, direct restorations using SFC either alone or as a bulk core in combination with conventional PFC composite show higher fracture resistance. In addition, the presence of SFC as a bulk core can reduce incidence of catastrophic failure compared to restorations without SFC.

## Data Availability

The data presented in this study are available on reasonable request from the corresponding author.
